# Supplementary material for: The Effects of Particulate Matter Alert on Urban Park Visitation in Seoul, Korea: Using Segmented Regression
Source: Int J Environ Res Public Health. 2022 Nov 21;19(22):15372. doi: 10.3390/ijerph192215372 (PMC9693038; doi:10.3390/ijerph192215372)
Supplement: Supplementary file 1 [file ijerph-19-15372-s001.zip › ijerph-1990972-supplementary.pdf]

Supplementary Material

The Effects of Particulate Matter Alert on Urban Park Visitation in Seoul, Korea: Using Segmented Regression

Yongsoo Choi [1], Garam Byun [2], Jong-Tae Lee [1,2,*]

[1] School of Health Policy and Management, College of Health Science, Korea University, 145, Anam-ro, Seongbuk-gu, Seoul 02841, Republic of Korea

[2] Interdisciplinary Program in Precision Public Health, Korea University, Seoul 02841, Republic of Korea

Correspondence: jtlee@korea.ac.kr; Tel.: +82-2-940-2770

Table S1. Standards and instructions for the PM_{10} alert system in Seoul.

		Alerts			
		Good	Normal	Bad	Very bad
Predicted PM_{10} concentration ($\mu g/m^3$)		0~30	31~80	81~150	Over 151
Advisory	Susceptible population*	-	No restrictions on outdoor activities. Nevertheless, be careful depending on your physical condition.	Limit prolonged or excessive outdoor activities. Asthmatics need to use the inhaler more frequently during outdoor activities.	Stay indoors if possible. Consult a doctor when doing outdoor activities.
	General population	-	-	Limit prolonged or excessive outdoor activities. Those with eye strain, cough or sore throat should avoid outdoor activities.	Limit prolonged or excessive outdoor activities. People with coughs or sore throats should avoid outdoor activities.

* Children, the elderly, and people with lung or heart disease

Table S2. Contingency table between the measured PM_{10} level and the PM_{10} alert results issued one day before afternoon (5 PM), Seoul, Korea, 2014-2019

		**Alert results (days, %)**				**sum**
		Good	Normal	Bad	Very bad	
**Measure PM_{10} level**	Good	273 (64.7)	149 (35.3)	0 (0)	0 (0)	422 (100)
	Normal	68 (6.4)	940 (88.1)	57 (5.3)	2 (0.2)	1067 (100)
	Bad	0 (0)	49 (45.4)	56 (51.9)	3 (2.8)	108 (100)
	Very	0 (0)	0 (0)	7 (70)	3 (30)	10 (100)
